# Enhancing emergency obstetric care navigation through a ‘Welcome Person’ model: insights from a health system strengthening initiative in Bangladesh

**DOI:** 10.7189/jogh.15.04128

**Published:** 2025-05-16

**Authors:** Hassan Rushekh Mahmood, Lubna Hossain, Farhia Azrin, Md Refat Uz Zaman Sajib, A K M Mahmudul Hassan, Trisha Mallick, Tanvir Hayder, Anisuddin Ahmed, Md Mehedi Hasan, Abu Sayeed, Sabrina Jabeen, Tajrin Tahrin Tonmon, Md Mahiur Rahman, Md Abu Bakkar Siddique, Shamsuz Zaman, Vibhavendra S Rasghuvanshi, Afruna Rahman, Haroon Bin Murshid, Nuzhat Nadia, Mustufa Mahmud, Md Azizul Alim, Shams El Arifeen, Dewan Md Emdadul Hoque, Abu Sayed Md Hasan, Ahmed Ehsanur Rahman

**Affiliations:** 1 Maternal and Child Health Division, International Centre for Diarrhoeal Disease Research, Bangladesh, Dhaka, Bangladesh; 2 Department of Health and Kinesiology, University of Illinois Urbana-Champaign, Urbana, Illinois, USA; 3 Global Health and Migration Unit, Department of Women's and Children's Health, Uppsala University, Uppsala, Sweden; 4 Poche Centre for Indigenous Health, The University of Queensland, Toowong, Queensland, Australia; 5 UNFPA, Dhaka, Bangladesh; 6 Infectious Diseases Division, International Centre for Diarrhoeal Disease Research, Bangladesh, Dhaka, Bangladesh; 7 Maternal, Newborn, Child & Adolescent Health, Directorate General of Health Services, Ministry of Health & Family Welfare of Bangladesh, Dhaka, Bangladesh; 8 UNICEF, Dhaka, Bangladesh

## Abstract

**Background:**

Maternal mortality remains critical in Bangladesh, driven by delays in accessing timely care at health care facilities. Globally, a woman dies every two minutes from pregnancy or childbirth, often due to systemic inefficiencies in health care. In Bangladesh, high maternal mortality rates are worsened by overcrowded facilities, limited resources, and complex procedures. The 'three delays' model identifies barriers to care, with the third delay – receiving timely treatment – being a major contributor to maternal deaths. This study aims to generate evidence on how the ‘Welcome Person’ can improve maternity care at the facility level in Bangladesh.

**Methods:**

We conducted a cross-sectional study from April to December 2022 among pregnant women at three selected health care facilities in Gaibandha District, Bangladesh. We deployed 20 ‘Welcome Persons’ to navigate and assist pregnant women, enhancing maternal health care. The Welcome Persons provided round-the-clock support, guiding mothers from the moment they entered the hospital through their admission, treatment, and any necessary referrals. The Welcome Persons maintained detailed time-stamped records, tracking patient movements and service timelines.

**Results:**

In this study of 5260 mothers, 47% presented with complications, with 52% arriving after office hours. The median time from entry to treatment was 15 minutes, with those without complications taking 14 minutes and those with complications 15 minutes. Entry-to-admission took a median of nine minutes, varying by age, with younger patients completing faster. Admission-to-treatment had a median time of six minutes, with severely complicated patients experiencing shorter times. Only 1% completed within five minutes, while 63% finished within 15 minutes. Upazila Health Complexes (UHCs) showed better performance in completing procedures within median times compared to the District Hospital (DH). Future study plans should include measuring maternal and neonatal outcomes as well.

**Conclusions:**

This study demonstrates that timely maternal care is achievable by deploying a support person. Using the ‘Welcome Person’ model to address admission bottlenecks, health care facilities can enhance patient experiences and outcomes. Despite a few limitations, evidence generated from this study can be utilised for scaling up decisions and can contribute to the health policy.

The Sustainable Development Goal (SDG) Target 3.1 aims to reduce the global Maternal Mortality Ratio (MMR) to no more than 140 per 100 000 live births by 2030 [1]. A 2023 recent report by United Nations (UN) agencies estimated that a woman dies every two minutes due to pregnancy or childbirth, with MMR either increasing or remaining stalled in many regions. This issue is particularly severe in low- and middle-income countries (LMICs), notably in sub-Saharan Africa and South Asia [2,3]. A critical barrier to reducing maternal mortality is the 'three delays' model:

1) delay in deciding to seek care

2) delay in reaching a health care facility

3) delay in receiving adequate care upon arrival [4,5].

The third delay is often due to systemic issues within health care systems, is particularly concerning and is a significant contributor to maternal mortality [6]. These systemic issues related to timely care at the facility constitute but are not limited to overcrowding, lower staff-to-patient ratios, absence of triaging [7] and poor patient navigation [8].

In Bangladesh, significant progress has been made in reducing maternal mortality however, the third delay remains a major challenge [9]. The 2016 Maternal Mortality and Health Care Survey (BMMS) reported an MMR of 196 (uncertainty range = 159–234) per 100 000 live births [10,11], with many maternal deaths linked to delays in receiving care. The survey revealed that 33% of women who died from maternal causes had sought care from multiple facilities, highlighting the impact of the third delay [11]. A quasi-experimental study conducted in four districts in Bangladesh in 2010 revealed that the median time required to obtain treatment at the health facility was 30-minute which is quite a long duration considering obstetric emergence [12].

Research in Bangladesh mentioned that limited patient understanding of health care systems, especially among rural or educationally disadvantaged women, insufficient communication with health care providers due to status barriers and cultural sensitivities, and limited patient-provider engagement can impede effective diagnosis and contribute to the third delay [13–15]. Factors such as low socioeconomic status, illiteracy, rural residency or distance of residence from the health facility, and poor communication between health care providers and patients exacerbate these delays which contribute to adverse outcomes from emergency medical conditions and even mortality [11,16,17].

In such a situation, patient navigation support can enhance the overall health care services and diminish inequalities for care-seeking pregnant women [18], as evinced by a previous study. Moreover, offering patient navigation services that are specifically customised to address the unique requirements of women could enhance maternity care [19]. Another study found that the 'Welcome Person’, a supporting cadre to guide and manage newborn cases and assist patients in navigating the health care system, can help minimise potential delays and enhance the quality of care [9]. Although patient navigation programmes and improved health care facility capacity have been proven helpful in prior studies, their implementation in women's health care has been less common compared to other patient populations [19–21]. Furthermore, there have been very few comprehensive investigations on the possibility of patient facilitation during the initial stage of accessing health care facilities to mitigate the third delay, especially in LMICs like Bangladesh.

This study investigates the impact of the 'Welcome Person' Model on reducing the third delay in maternity care services in Bangladesh. We hypothesise that deploying a designated 'Welcome Person' would assist women in navigating the health care system upon their arrival at the health facility and enhance their access to services and receive appropriate and timely care, enhance communication between patients and health care providers, and improve patient satisfaction with their health care experiences. This paper aims to generate evidence on the role of a ‘Welcome Person’ in enhancing maternity care at the facility level in Bangladesh who encounter comparable difficulties.

## METHODS

### Study design and site

We conducted a cross-sectional study from April 2022 to December 2022 among pregnant women in selected three health care facilities from Gaibandha district which was one of the eight selected districts under the ‘Better Health District Model’ project. Gaibandha district is under the Rangpur Division is located in the Northern part of Bangladesh. In Gaibandha district, the study sites were Gaibandha District Hospital (DH), and two sub-district hospitals/Upazila Health Complexes (UHCs) (Sadullapur UHC and Gobindaganj UHC) of Gaibandha district. These public health care facilities were selected based on recommendations from the Maternal, Newborn, Child, and Adolescent Health (MNC & AH) programme of the Directorate General of Health Services (DGHS) of Bangladesh (**Table 1**).

**Table 1:** Basic information of study area from DHIS2*

Sl No.	Facility name	Basic Information				
**District name**	**Division name**	**Total delivery (2022)**	**Pregnant women with complication (2022)**
1	Gaibandha DH	Gaibandha	Rangpur	1234	892
2	Sadullapur UHC	Gaibandha	Rangpur	623	188
3	Gobindaganj UHC	Gaibandha	Rangpur	1829	489

DH – District Hospital, DHIS2 – District Health Information System, version 2, UHC – Upazila Health Complex

*Source: [22].

The ‘Better Health District Model’ project had multiple components aimed at enhancing the quality of maternal health care. These components included ensuring 24/7 Emergency Obstetric and Newborn Care (EmONC) services, Robson classification implementation, comprehensive EmONC and Robson classification training, regular monitoring and supervision of services, and ongoing capacity development programmes for health care providers. In addition to these initiatives, the project introduced the ‘Welcome Person’ intervention to facilitate patient care.

### Study population

All the mothers irrespective of their age, gestational age and complication who came for antenatal care (ANC), delivery and/or postnatal care (PNC) in the mentioned three health facilities during the study period were enrolled in this study.

### Welcome person intervention

This project aimed to ensure 24/7 Emergency Obstetric and Newborn Care (EmONC) services and to enhance high-quality maternal health care through implementing an intervention package. One of the key components of the intervention package was ‘Welcome Person’. A total of 20 ‘Welcome Person’ were deployed in these three facilities, aiming to support pregnant women in the hospital at every step to ensure timely health care services at the facilities, starting from entry to the emergency department or the outdoor/reception of the facility and to all other places like labour ward/room, operation theatre complex where pregnant women may need to seek services. Eight were placed in DH and six in each UHC. They were on daily roster duty round the clock. The number of welcome persons was higher in the morning shifts (n = 4) than in the evening (n = 2) and the night shift (n = 1), keeping in mind the general patient flow in the DH. The other had a day off (last duty on the previous day) (Figure S1 in the **Online Supplementary Document**). At UHC, three welcome persons were assigned to the morning shift, one to the evening shift, and the night shift and day off followed the same pattern as the DH. This study focused on investigating outcomes in three mentioned health facilities where the 'Welcome Person' model was initiated. Prior to their deployment, a day-long extensive training was conducted that included guidance regarding the facilitation, posing no risk to mothers, and overcoming other care-seeking barriers like language and communication with health care providers. In addition, to retain their competency, quarterly refresher training was arranged for them as well.

The ‘Welcome Person’ facilitated/navigated the patients mainly in four ‘points of care’:

1) Emergency and Obs-Gynae outdoor: Upon arrival of the pregnant woman at the emergency department/Obs-Gynae outdoor of the mentioned facilities, the on-duty welcome person greeted and navigated her to the emergency department/Obs-Gynae outdoor. Then he informed the on-duty doctor and/or the on-duty nurse of the emergency department/Obs-Gynae outdoors about the pregnant mother.

2) Admission: After completing the initial evaluation of the pregnant mother, if she was advised to be admitted to the labour ward, the welcome person navigated the pregnant mother and her companion to the admission counter and facilitated them to complete the admission process with priority. If admission was not advised, they were directed to the appropriate facility point (outdoor/exit) based on their needs.

3) Navigation to labour room/labour ward/operation theatre: After completing the admission process, the welcome person then asked the pregnant mother about her choice of navigating: either she needed a patient stretcher or wheelchair or she could go walking. Depending on the mother's choice, the welcome person navigated her to the labour ward, ensuring her comfort. Reaching the labour ward, the welcome person then informs about the newly arrived patient to the on-duty doctor or on-duty health care professionals, which in this case were either Senior Staff Nurses or Midwives. Then, the welcome person carefully facilitated the pregnant mother to reach her assigned bed. They also provided necessary support to the pregnant mother in reaching the labour room/operation theatre and guided her if she needed to visit the facility pharmacy for medication or the laboratory for necessary investigations.

4) Referral: If a pregnant mother was referred to a higher facility, the welcome person promptly facilitated the transfer of the mother from the emergency/ outdoor/labour ward/labour room/operation theatre to the referred facility by actively supporting the patient’s attendants in calling the ambulance and pushing the patients’ stretcher/wheelchair towards the ambulance. Additionally, when pregnant women or recently delivered mothers were referred from Sadullapur and Gobindaganj UHC to Gaibandha DH, the welcome person informed the DH’s respective welcome person prior to the patient's arrival, allowing them to prepare by arranging a patient’s stretcher or wheelchair and taking other necessary steps.

**Figure 1:**
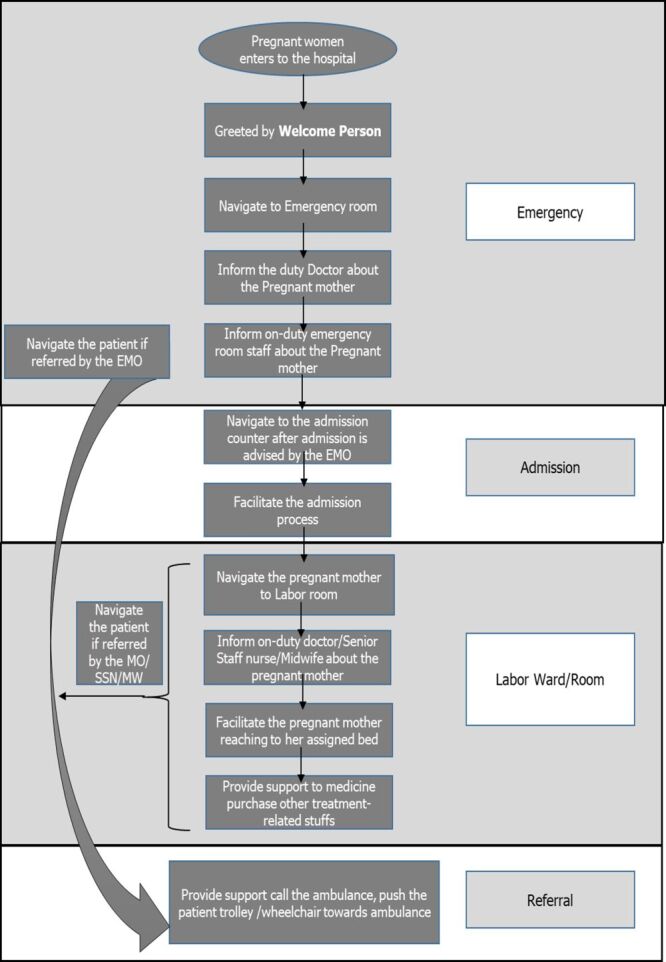
Process flow of patient facilitation/navigation by the ‘Welcome Person’ at the facilities.

The following activities were facilitated by the welcome person at the facilities (**Figure 1**).

### Data collection

Data were collected over nine months from the mentioned three facilities (one DH and two UHCs). Time stamping (recording a particular time) was ensured by the on-duty Welcome Person at different ‘points of care’ by filling up a structured record-keeping tool (‘Patients’ register) developed by the investigators’ team. The register comprised particulars of the patient/mother, mode of entrance in the hospital, particulars of arrival, time of procedure completion at different points of care (entry to the hospital, admission and treatment start), presence of any maternal complication (haemorrhage, eclampsia, obstructed labour, *etc*.) *etc*. Gravity of complication (not complicated, mild complicated and severe complicated) data were also recorded based on the assessment by the health care providers in those hospitals. Complications were categorised into three mutually exclusive groups based on the severity. Those who presented for delivery without any complications were classified as ‘not complicated.’ Patients who experienced any complications during pregnancy, delivery, or after pregnancy were considered ‘mildly complicated.’ Finally, patients who arrived at the facility in a critical condition, such as unconscious or semi-conscious, or with severe issues like excessive bleeding, convulsions, or labour lasting more than 12 hours, were categorised as ‘severely complicated’. A field supervisor was in charge of monitoring and ensuring quality service provided by the ‘Welcome Person’ as well as the accurate time stamping/record keeping.

### Ethical considerations

The Institutional Review Board of icddr,b granted ethical permission for the study protocol (PR-23075), and the Maternal Health programme of MNC & AH, Directorate General of Health Services (DGHS) granted administrative approval. Prior to recording the above-mentioned data of the mothers, signed informed consent was obtained from each of the mothers by the Welcome Person. Illiterate respondents were requested to verbally assent, and their thumbprints were taken. In case of severe illness, informed consent was obtained from the attendants.

### Statistical analysis

To check the normality of the data, we have plotted histograms for mothers’ entry in the hospital to starting of the treatment at the indoor (whole procedure), entry in the hospital to completion of the admission process (entry to admission), and completion of the admission process to starting of the treatment at the indoor (admission to starting treatment) using STATA, version 15 (StataCorp LLC, College Station, Tx, USA). Additionally, we have done a Shapiro-Francia normality test to check the normality of the data. The test reflects that our data are not normally distributed thus, we used the median for our analysis. We have explored the background characteristics of the patients, including their level of complication (not complicated, complicated, or severely complicated), type of facility (DH, UHC), time of arrival (office hours = 8 am–2 pm; after office hours = 2 pm – 8 am), day of arrival (weekdays, weekend), age (<20 years, 20–34 years, 35–49 years), way of arrival (walking, not walking), and quarters (Q1 = April–June; Q2 = July–September; Q3 = October–December). Our main outcome of interest was the duration of time of the initial assessment at the emergency/OPD to start the treatment procedure, initial assessment at the emergency/OPD to admission procedure, and admission to start the treatment procedure. We explored the distribution of the mentioned time duration in different categories.

## RESULTS

### Patient demographics

A total of 5260 mothers were enrolled for this study, and almost half of them (47%) came to the hospital having any maternal complications (**Table 2**). Among all the patients, most of them (52%) came after office hours than during office hours. The majority (72%) of the patients were aged between 20 and 34 years, and most (90%) of the patients arrived by walking from facility entry point to the other point of care.

**Table 2:** Pregnant women attended by the welcome person in Gaibandha (April 2022–December 2022)

Variable	Category	Frequency, n = 5260	Percentage (%)
Complication	Not complicated	2385	45
	Moderately complicated	1814	35
	Severely complicated	636	12
	Missing	425	8
Facility	DH	2308	44
	UHC	2952	56
Time of arrival	Office hour	2513	48
	After office	2747	52
Day of arrival	Weekdays	4662	89
	Weekend	598	11
Age	<20 y	1056	20
	20–34 y	3774	72
	35–49 y	380	7
	Missing	50	1
Way of arrival	Not walking	505	10
	Walking	4738	90
	Missing	17	0
Quarter	Q1 (April–June)	1246	24
	Q2 (July–Sept)	1864	35
	Q3 (Oct–Dec)	2150	41

DH – District Hospital, UHC – Upazila Health Complex

### Median time metrics

**Figure 2:**
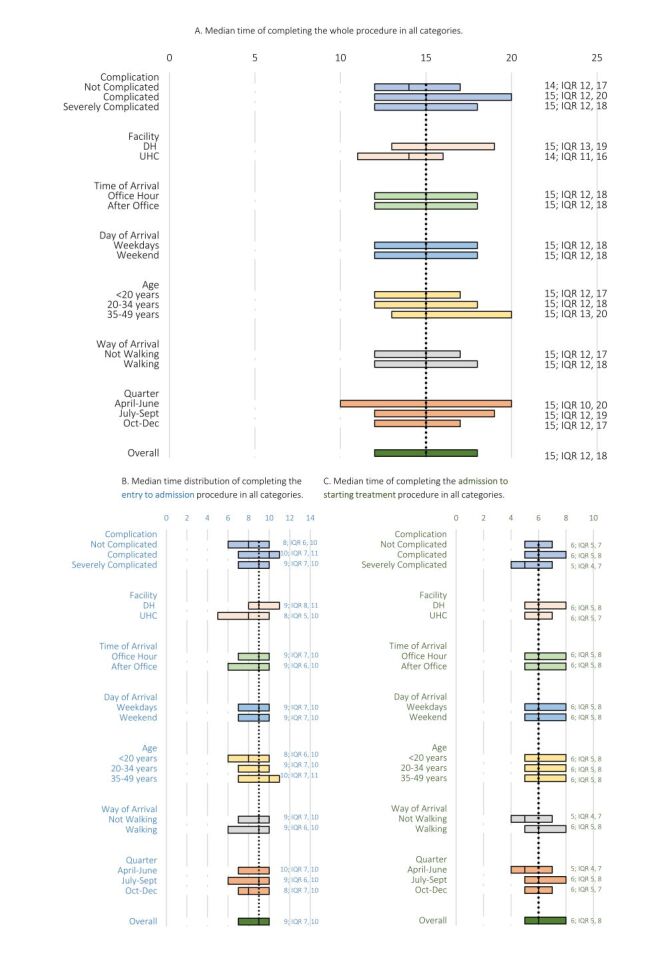
Median time of completing the procedure in all categories. **Panel A.** Median time of completing the whole procedure in all categories. **Panel B.** Median time distribution of completing the entry to admission procedure in all categories. **Panel C.** Median time of completing the admission to starting treatment procedure in all categories.

The overall median time for entry to starting treatment (whole procedure) was 15 minutes. Patients without complications had a shorter median time (14 minutes) from entry to starting of the treatment compared to the median time (15 minutes) of patients with complications (**Figure 2**, Panel A).

The median time from entry to admission procedure was nine minutes (interquartile range (IQR) = 7–10 minutes) and varied by age. More than 50% of patients aged less than 20 years completed the admission within eight minutes, while more than half of the patients aged more than 34 years completed the procedure in 10 minutes (**Figure 2**, Panel B).

The median time from admission to starting treatment was six minutes (IQR = 5–8 minutes). Severely complicated patients had a shorter median time (five minutes) to start treatment than the median time (six minutes) of those with mild or no complications (**Figure 2**, Panel C).

**Figure 3:**
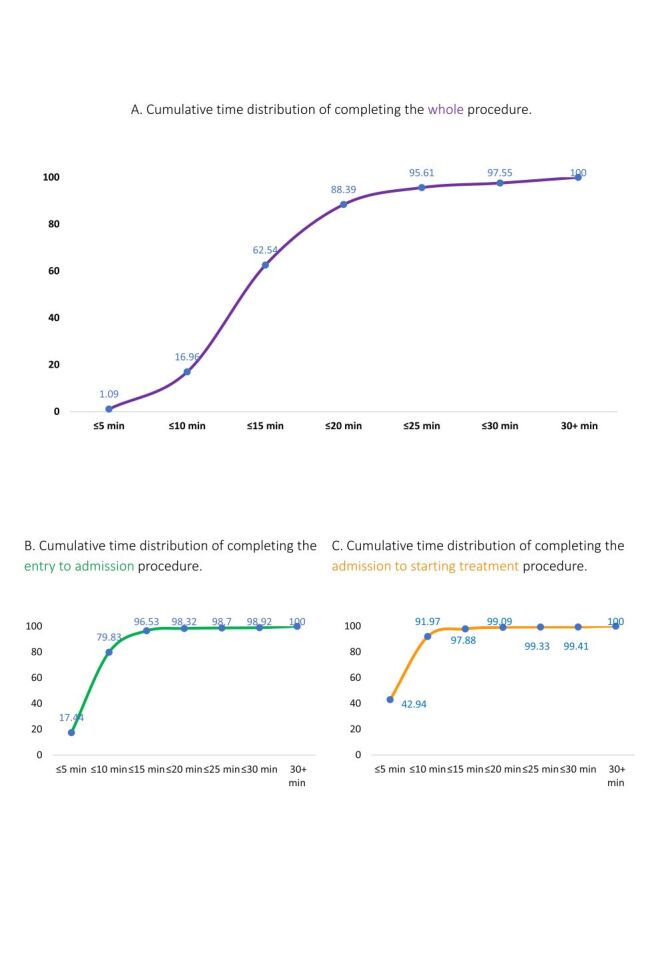
Cumulative time distribution of completing the procedure. **Panel A.** Cumulative time distribution of completing the whole procedure. **Panel B.** Cumulative time distribution of completing the entry to admission procedure. **Panel C.** Cumulative time distribution of completing the admission to starting treatment procedure.

Only 1% of the patients completed the whole procedure within 5 minutes, while nearly 63% finished within the median time (15 minutes) (**Figure 3**, Panel A). From the entry-to-admission procedure, the overall median time was nine minutes, with almost 80% completed within 10 minutes (**Figure 3**, Panel B). For admission-to-starting the treatment procedure, the overall median time was six minutes, with over 90% completing within 10 minutes (**Figure 3**, Panel C).

Among the pregnant women who completed the whole procedure, only 0.80% of severely complicated and 1.70% of complicated patients took less than or equal to five minutes to complete the whole process. On the other hand, two-thirds of the patients (66.92%) with no complications completed the procedure within 15 minutes (median time), while around 57% of patients with severe complication completed the procedure within the median time (Figure S2 in the **Online Supplementary Document**).

### Facility comparisons

Comparing among the facilities, the proportion of patients who completed procedure within the median time was higher for UHC compared to DH. Only 52.16% of patients in DH completed the procedure in the median time of 15 minutes, while 71.47% of UHC patients completed the whole procedure in 15 minutes (Figure S3 in the **Online Supplementary Document**). More than 60% of patients completed the whole procedure within the median time (15 minutes) regardless of arriving time and on weekdays or weekends. Patients who arrived at the facility by walking and those who came by wheelchair, patient’s stretcher, *etc*. for both of these two categories, almost two-thirds of patients completed the procedure within the median time.

Of mothers who completed entry to admission procedure, 22.07% of non-complicated and 13.06% of complicated patients took less than or equal to five minutes to complete the whole process. The admission completion varied between DH and UHC. In UHC, over one-fourth of the patients completed the admission within 5 minutes, compared to only 7% within five minutes at DH.

Among the pregnant women who completed the admission to start the treatment procedure, more than 50% with severe complications completed the procedure within five minutes, while the proportion was around 40% for not complicated patients. In UHC, the rate of starting treatment within 5 minutes was higher compared to the patients in DH.

## DISCUSSION

This study sheds light on critical aspects of maternal care delivery by examining the usefulness of patient facilitation/navigation by 'Welcome Person' in reducing the third delay in Bangladesh. Delay in receiving appropriate care at health care institutions is a major factor in maternal mortality rates, especially in LMICs like Bangladesh. By examining the patient journey from entry to treatment initiation, the study provides valuable insights that can inform policy and practice. The intervention explored that the patients required 15 minutes as an overall median time for arrival at the hospital to the start of the treatment. The intervention required minimal technical training, monitoring and suggested the potential for wider implementation in similar settings.

In Bangladesh public health facilities, there was no such dedicated support workforce specific to maternal health care. To address this gap, 'Welcome Persons' were introduced to guide pregnant women throughout the hospital, enhancing maternal health care services. This initiative was implemented in one district hospital and two sub-district hospitals, representative of similar health care settings. One previous study on syndromic sepsis management mentioned a median value of 35 minutes to start treatment after arriving in the emergency department, with an IQR of 25–45 minutes [9]. An operation research on ‘safe motherhood’ revealed that the evaluation interval (time to arrival at the hospital to evaluation by a professional that follows treatment start) averaged 30 minutes across countries [23]. The ‘Welcome Person’ intervention revealed a median time of 15 minutes from the entry to starting the treatment. This duration reflects the efficiency of the health care system in promptly attending to patients upon arrival.

Among the participants, nearly half (45%) of the pregnant women who visited these facilities had no complications, while only about one-tenth had severe complications. Interestingly, those without any maternal complications had a shorter median time from entry to the start of treatment. This finding suggests that streamlined processes and effective patient navigation are crucial in expediting care. Patients with severe maternal complications justifiably require more time due to the intricacy of their conditions. To address these findings, speeding up entry and admission for patients without complications and ensuring adequate support for those with complicated cases could be helpful. Regularly reviewing and improving patient guidance and care could also enhance overall efficiency.

Our findings on enhancing the promptness of maternal care access by the ‘Welcome Person’ are consistent with other research showing patient navigation can reduce disparities in obstetric and benign gynaecological care [19]. Moreover, evidence showed that the presence of such a support cadre, like a 'Welcome Person', can effectively reduce any possible delays and improve the overall quality of health care [9]. Conversely, a systematic review of patient navigation programmes for adult cancer patients revealed no significant impact on the quality of life and distress levels of the patients but did show a statistically significant improvement in patient satisfaction [24].

While global trends align with the study’s findings, Bangladesh’s context introduces unique challenges, such as population density, resource constraints, and cultural norms influencing maternal care. The ‘Welcome Person’ intervention demonstrates its effectiveness by the median time of nine minutes from arrival to admission and has the potential to create an impact on the patient’s experience of care. The median time for uncomplicated pregnant women to complete the admission process was eight minutes, two minutes faster than those with any maternal complications and one minute faster than those with severe complications. Also, the sub-district hospitals were quicker, with an eight-minute median time from arrival to admission, compared to nine minutes at district hospitals, likely due to lower patient volumes. Thus, there might be scalability challenges for districts or higher facilities. However, studies on a larger scale can generate further evidence to explain this hypothesis.

The median time between hospital; entry to admission (nine minutes) and admission to starting treatment (six minutes) highlights several areas of optimisation. Streamlining the admission process by enhancing registration, assessment, triage, and bed allocation can further reduce the overall care delivery time. Additionally, the ‘Welcome Person’ plays a crucial role in guiding patients, assisting with paperwork, providing orientation, and conducting initial assessments. Expanding this intervention could significantly enhance the care delivery process and lead to even better outcomes.

The ‘Welcome Person’ intervention was to provide 24/7 additional facilitation support to expectant mothers who might otherwise avoid district hospitals or government facilities due to delayed and complicated services-seeking procedures. The ‘Welcome Person’s’ constant presence facilitated navigation, reduced the influence of unwanted brokers, and addressed a critical preparedness gap in the Bangladeshi health care system- a dedicated support cadre for assisting these vulnerable care-seekers. Moreover, this novel approach requires no technical and in-depth training other than the guidance regarding the facilitation, poses no risk to mothers, and overcomes other care-seeking barriers like language and communication with health care providers in local areas.

The intervention, if conducted independently and considering sustainability, would require additional funding for extensive research considering patient flow across different areas (locality-wise) and types of health care facilities (tier-wise). Effective communication about the intervention’s importance can change the perception of the local stakeholders along with the families of the pregnant women. Moreover, a national collaboration of government and non-government entities is crucial for such interventions that require multi-level adaptations, including human resources retention and organisational structures. Despite its small-scale implementation, we may still apply the lessons learned to scale up, potentially reducing maternal mortality by addressing the delay in receiving care at the health facility. Conducting implementation research in other LMICs can generate further evidence that may strengthen the policy and practice globally.

The project successfully addressed the needs of all pregnant women accessing the included facilities; however, it posed some implementation-related limitations. First, the lack of prior situation analysis and experimental design limits the precision and effectiveness of the intervention and misses the generation of critical insights. Additionally, the recruitment of local residents as ‘Welcome Person’ leaves the possibility of some reporting bias. Moreover, for security concerns, we could not assign female ‘Welcome Person’ during evening and night shifts, potentially impacting the overall effectiveness of the intervention, as patients may feel more comfortable receiving assistance from their own sex. Furthermore, our study revealed a distinct occurrence of digit preference among the ‘Welcome Person’ who did the time stamping (numerical data). This could create additional bias and affect the accuracy of findings. Mixing of local hire and project-supported staff, pairing a female ‘Welcome Person’ with a male during the night shift, and enhancing monitoring targeting digit preference could be the possible solutions to overcome these limitations. Future research should take appropriate caution for such inclination and contemplate methods to alleviate its influence.

## CONCLUSIONS

The study demonstrates that timely maternal care is achievable in Bangladesh through the implementation of a support person though this study lacks proving ‘Welcome Person’s’ role as a sole driver for improving time metrics. However, by adopting the ‘Welcome Person’ model and addressing bottlenecks in the admission process, health care facilities can enhance patient experiences and outcomes. This, as a broader outcome, can contribute to the national health policy of Bangladesh and also can address sustainable development goals (SDG, goal-3.1). Future research should explore the impact of these interventions on maternal and neonatal outcomes considering patient flow in different localities and settings as well as tiers of health facilities inclusive of cost implication and scalability.

## Additional material


Online Supplementary Document
