# Supplementary material for: Enhancing emergency obstetric care navigation through a ‘Welcome Person’ model: insights from a health system strengthening initiative in Bangladesh
Source: J Glob Health. 2025 May 16;15:04128. doi: 10.7189/jogh.15.04128 (PMC12082253; doi:10.7189/jogh.15.04128)

Supplement to: Mahmood HR, Hossain L, Azrin F, Sajib MRUZ, Hassan AKMM, Mallick T, Hayder T, Ahmed A, Hasan MM, Sayeed A, Jabeen S, Tonmon TT, Rahman MM, Siddique MAB, Zaman S, Rasghuvanshi VS, Rahman A, Murshid HB, Nadia N, Mahmud M, Alim MA, Arifeen SE, Hoque DME, Hasan ASM, Rahman AE. Enhancing emergency obstetric care navigation through a 'Welcome Person' model: insights from a health system strengthening initiative in Bangladesh. J Glob Health. 2025;15:04128.

Supplementary Figure

Figure S1: Welcome Person shift duty and distribution

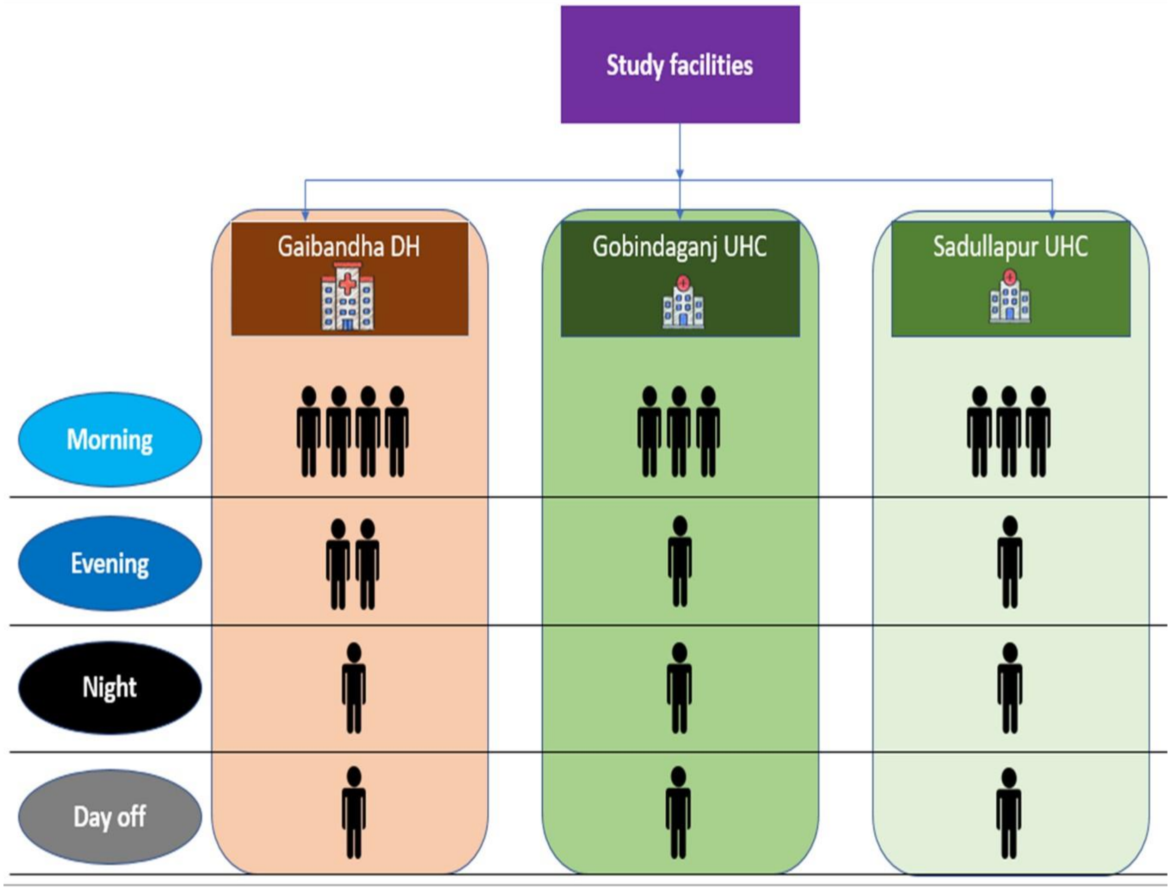

Figure S2: Cumulative time distribution of completing the whole procedure by complication level.

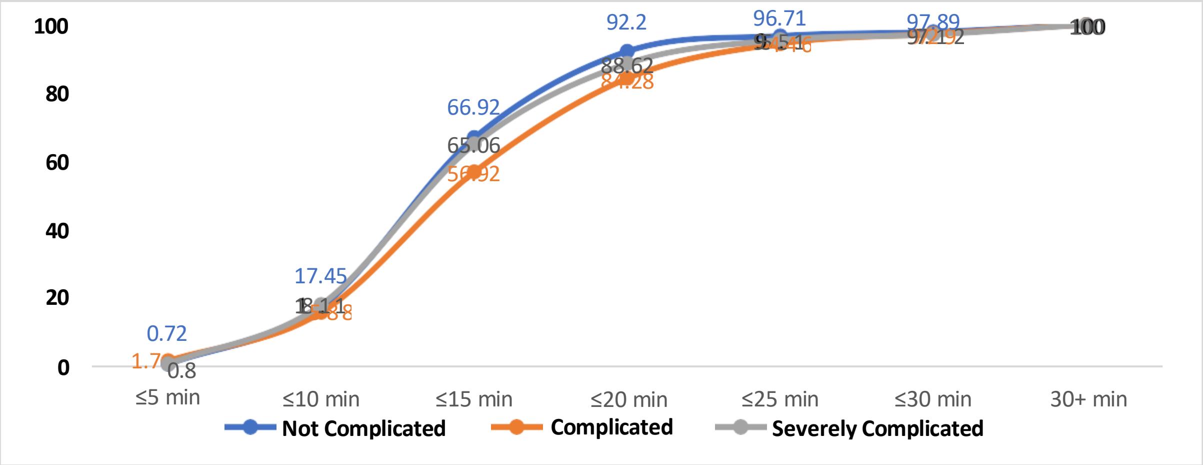

Figure S3: Cumulative time distribution of completing the whole procedure by facility level.

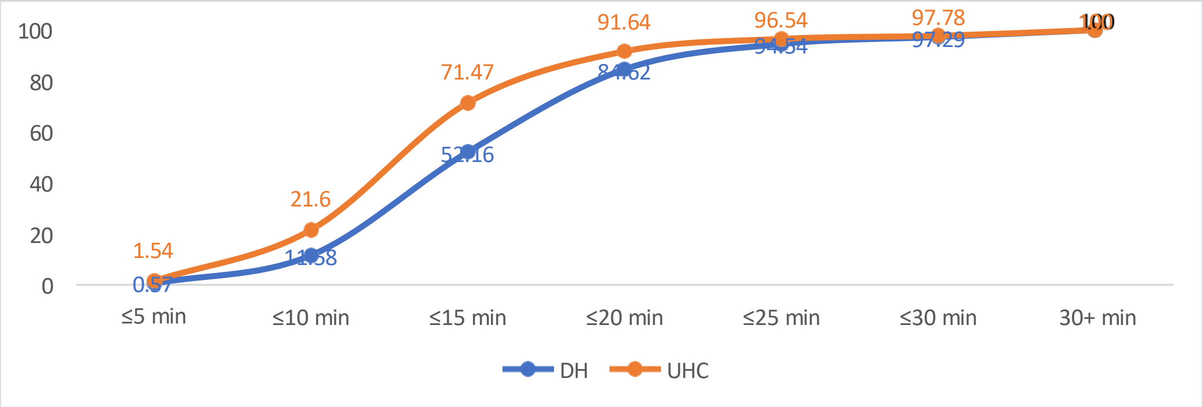

Figure S4: Cumulative time distribution of completing the whole procedure by time of arrival.

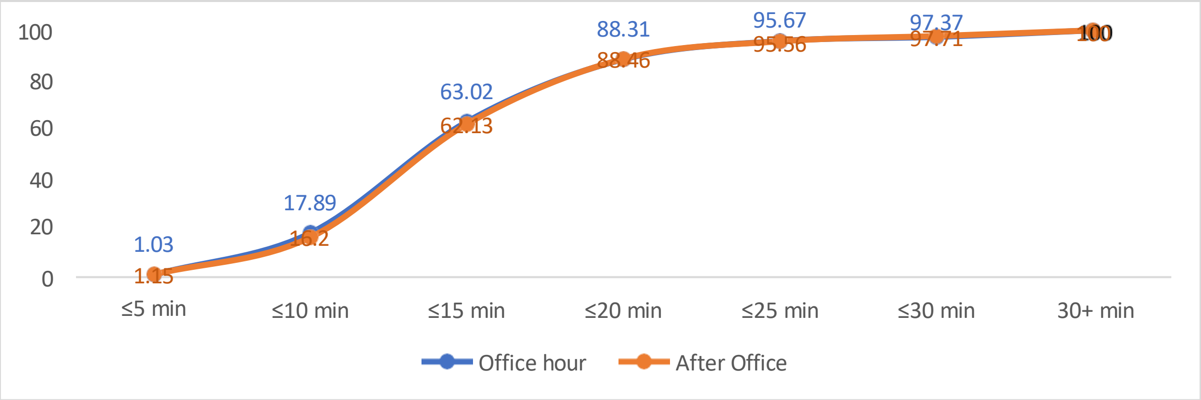

Figure S5: Cumulative time distribution of completing the whole procedure by day of arrival.

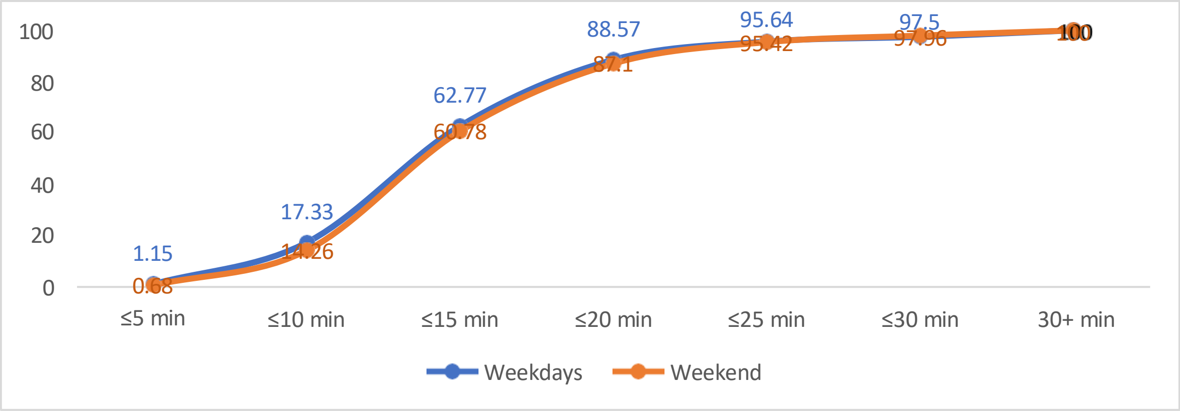

Figure S6: Cumulative time distribution of completing the whole procedure by age distribution.

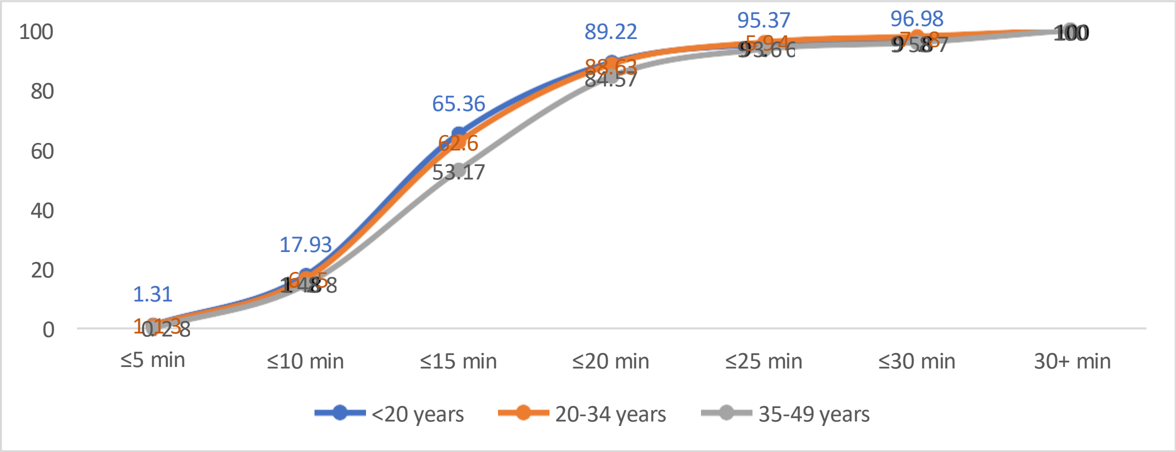

Figure S7: Cumulative time distribution of completing the whole procedure by way of arrival.

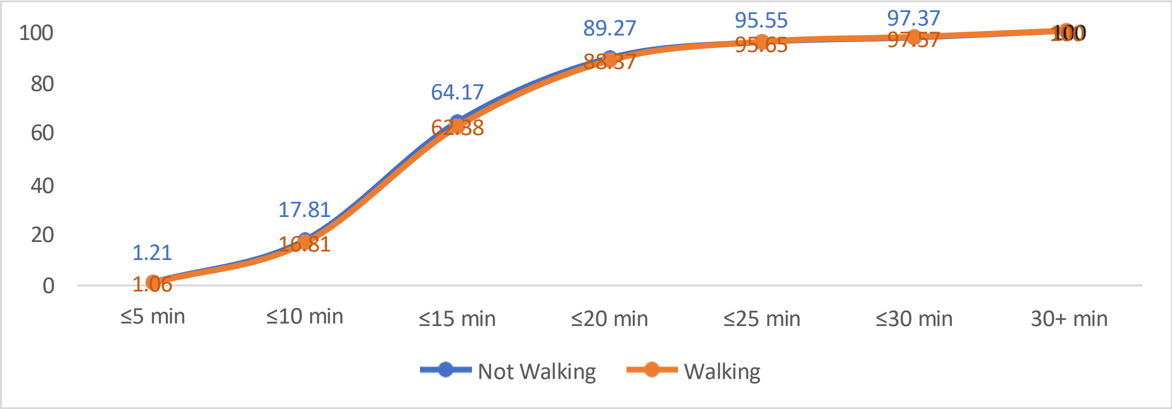

Figure S8: Cumulative time distribution of completing the whole procedure by quarter.

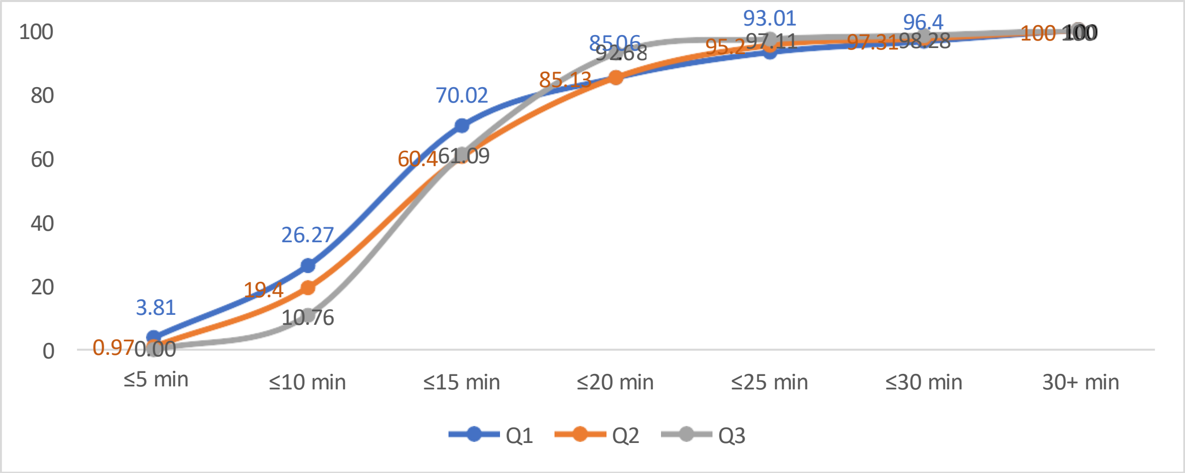

Figure S9: Cumulative time distribution of completing the entry to admission procedure by complication level.

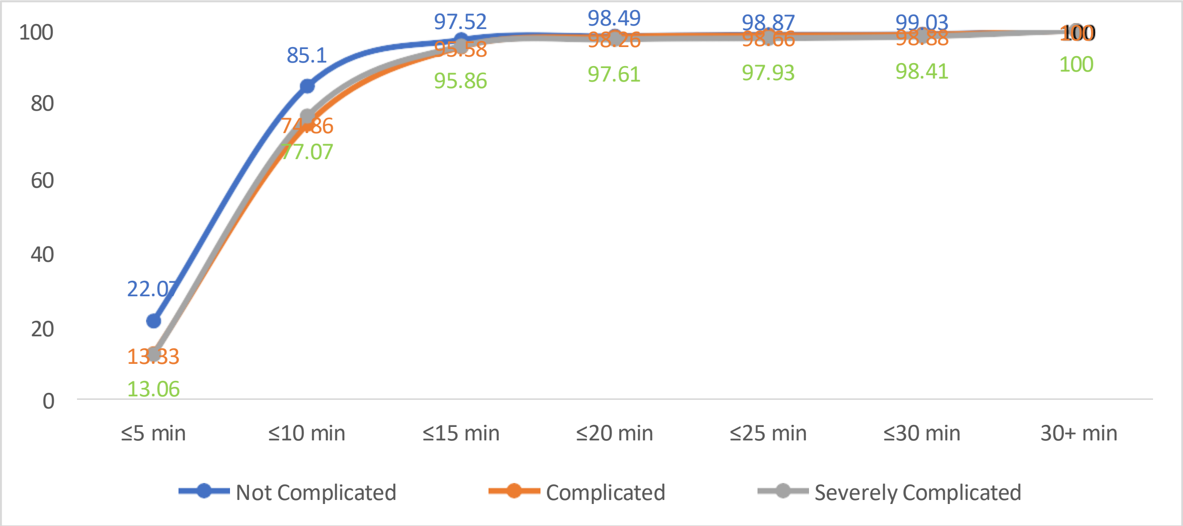

Figure S10: Cumulative time distribution of completing the entry to admission procedure by facility level.

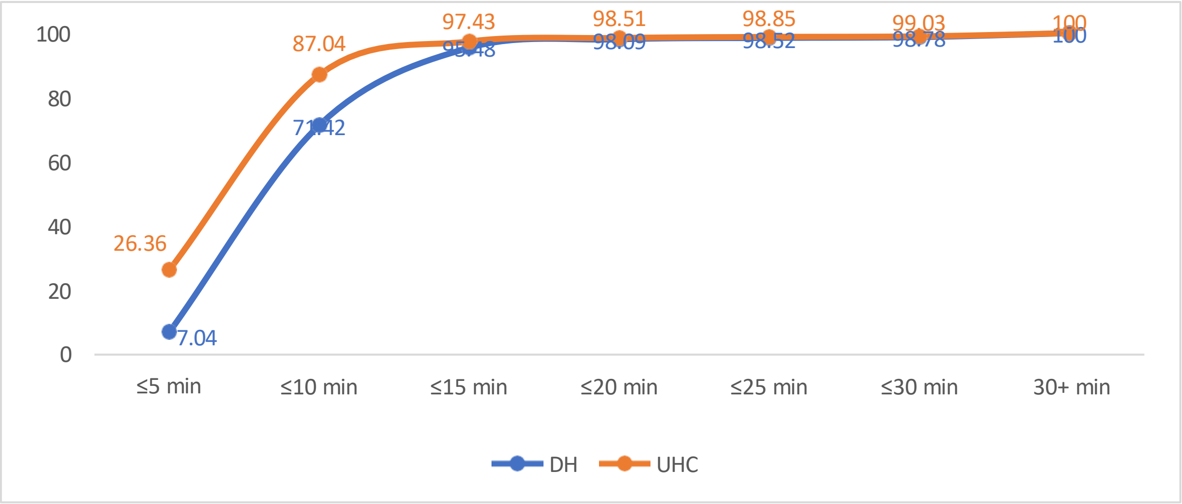

Figure S11: Cumulative time distribution of completing the entry to admission procedure by time of arrival.

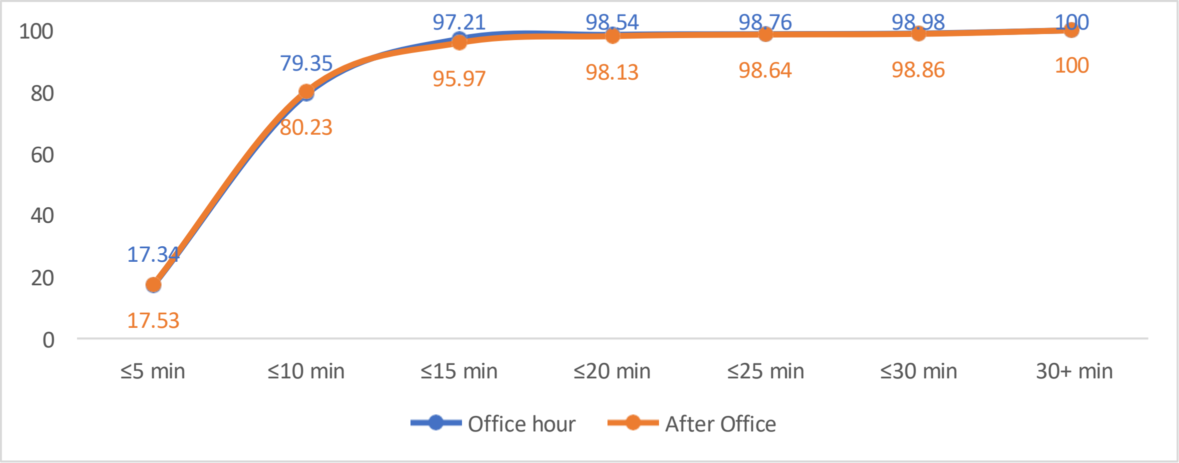

Figure S12: Cumulative time distribution of completing the entry to admission procedure by day of arrival.

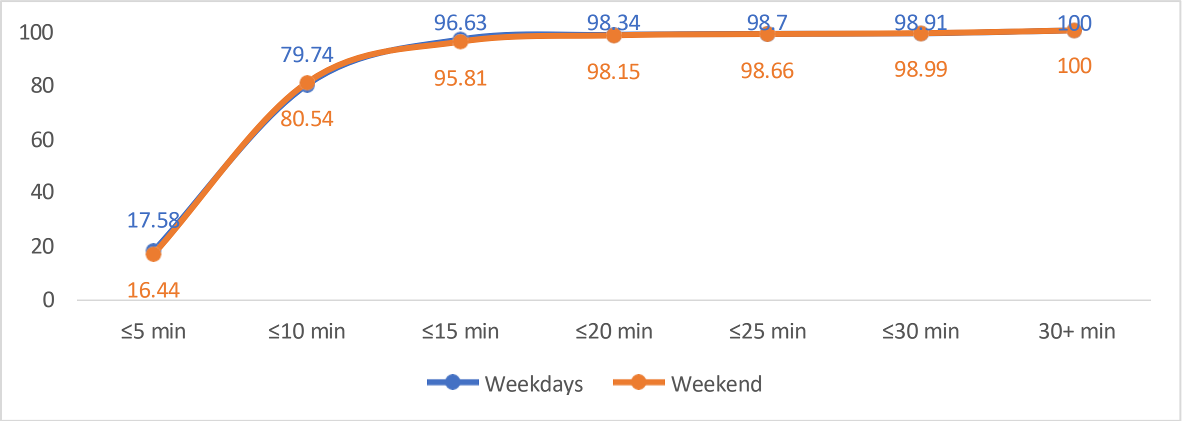

Figure S13: Cumulative time distribution of completing the entry to admission procedure by age distribution.

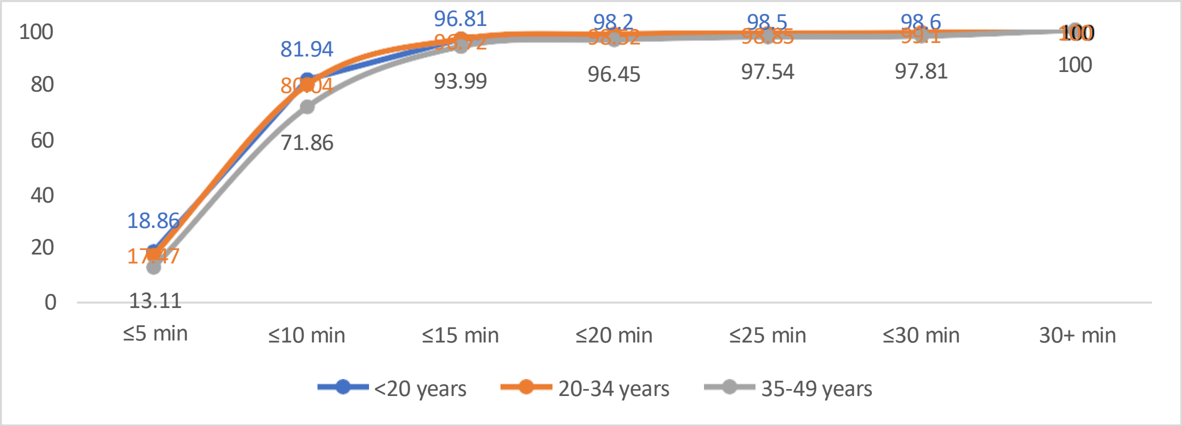

Figure S14: Cumulative time distribution of completing the entry to admission procedure by way of arrival.

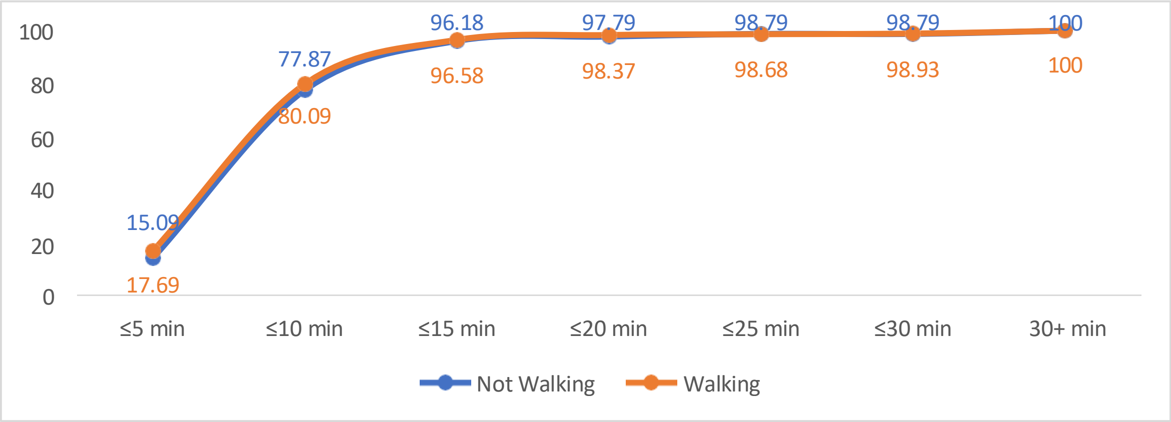

Figure S15: Cumulative time distribution of completing the entry to admission procedure by quarter.

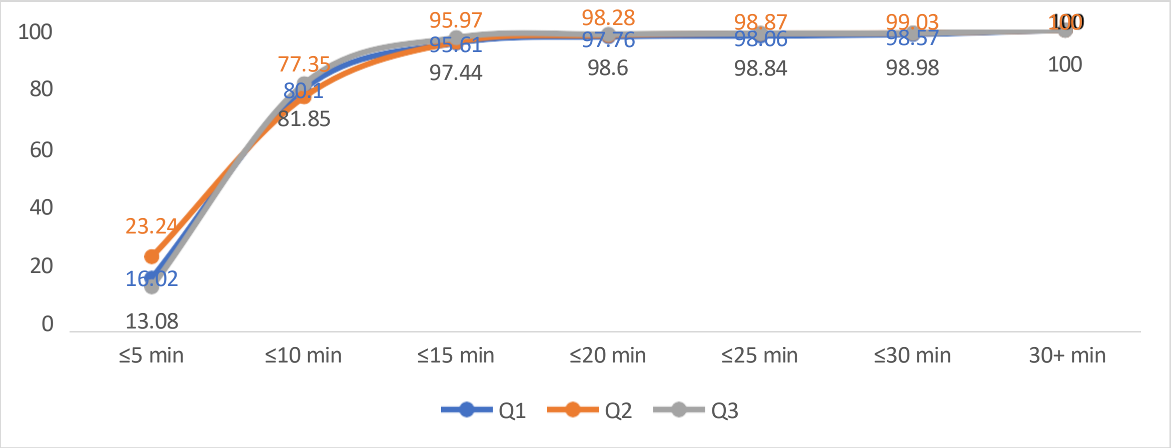

Figure S16: Cumulative time distribution of completing the admission to starting of treatment procedure by complication level.

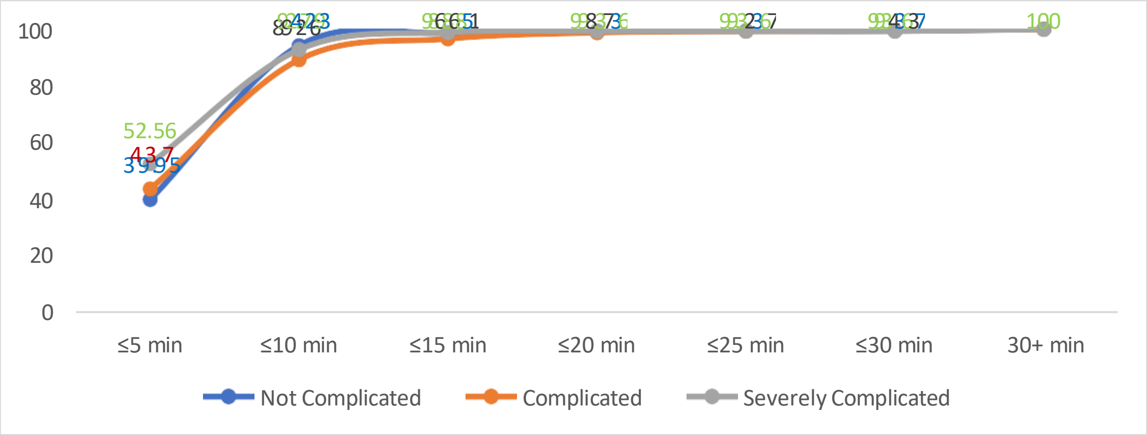

Figure S17: Cumulative time distribution of completing the admission- starting treatment procedure by facility level.

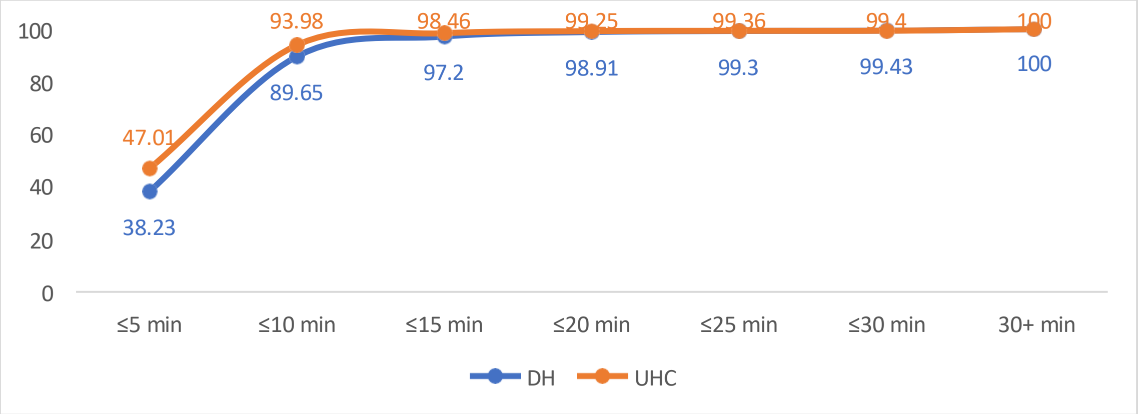

Figure S18: Cumulative time distribution of completing the admission- starting treatment procedure by time of arrival.

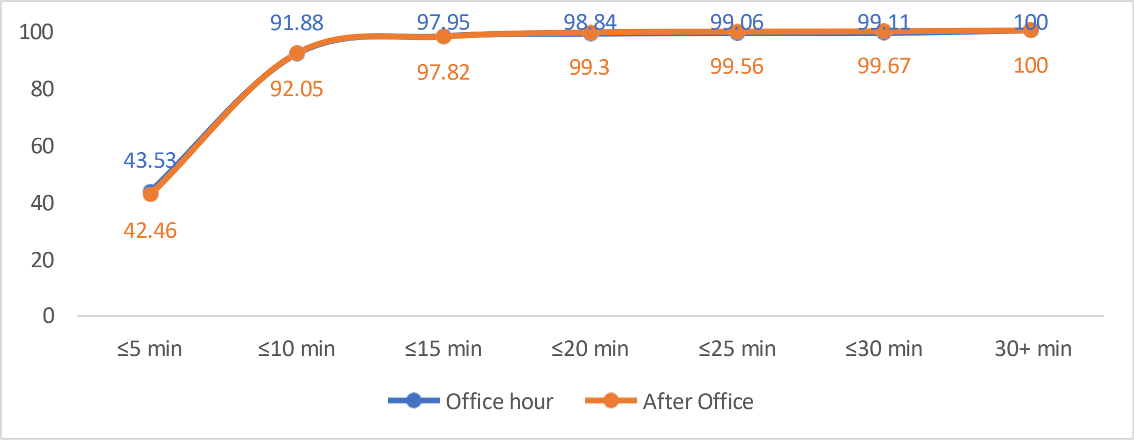

Figure S19: Cumulative time distribution of completing the admission- starting treatment procedure by day of arrival.

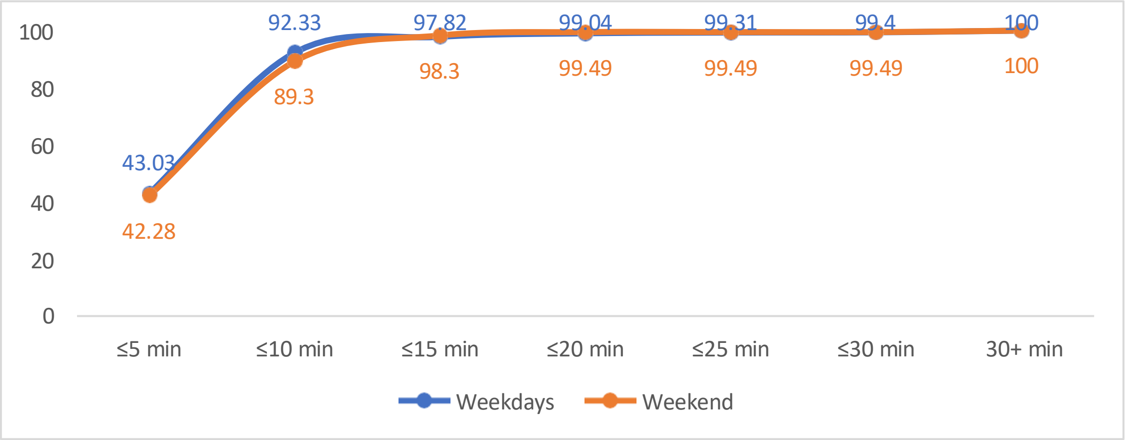

Figure S20: Cumulative time distribution of completing the admission to starting treatment procedure by age distribution.

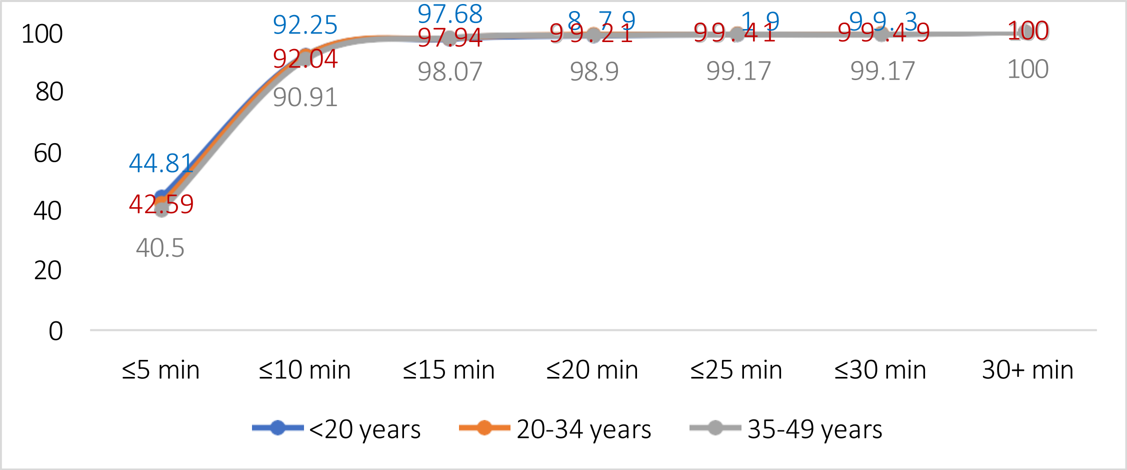

Figure S221 Cumulative time distribution of completing the admission- starting treatment procedure by way of arrival.

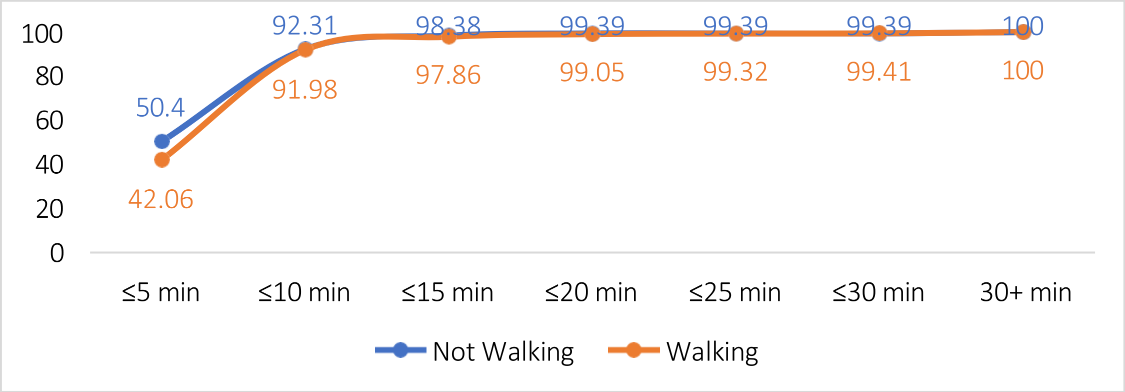

Figure S22: Cumulative time distribution of completing the admission- starting treatment procedure by quarter.

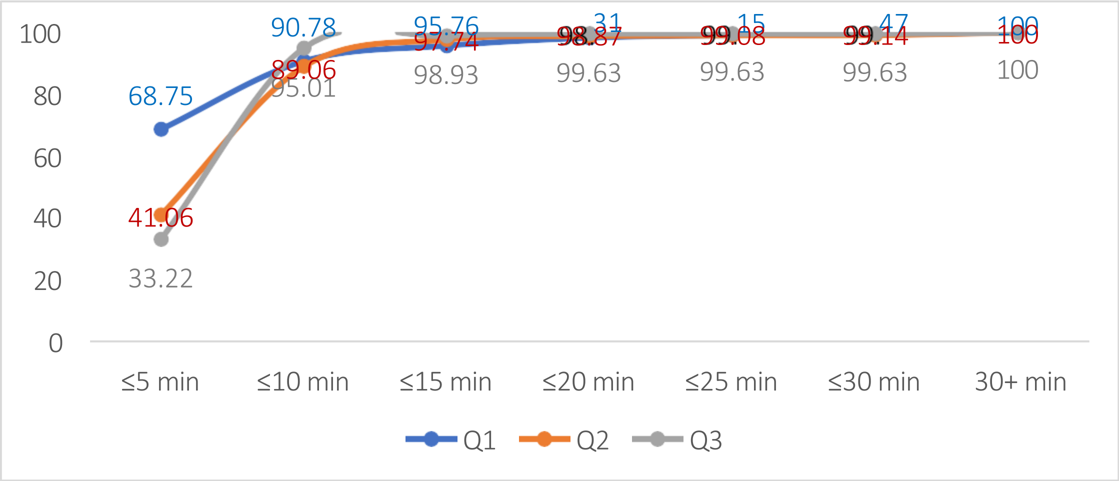

Supplement: Online Supplementary Document [file jogh-15-04128-s001.pdf]
